# Mommy, Can I Play Outside? How Urban Design Influences Parental Attitudes on Play

**DOI:** 10.3390/ijerph20064909

**Published:** 2023-03-10

**Authors:** Jinyun Lyu, Huiying Yang, Stella Christie

**Affiliations:** 1 Tsinghua Laboratory of Brain and Intelligence, Tsinghua University, Beijing 100084, China; 2 Department of Psychology, Tsinghua University, Beijing 100084, China

**Keywords:** free play, learning, urban design, parental beliefs, child-friendly cities

## Abstract

Although play results in physical, social, and cognitive benefits, there is a consensus that children’s opportunities to play have been reduced, particularly for those who live in urban environments. What are the barriers to play, and how can we mitigate them? This review examines a critical factor in play opportunities: parents as the decision-makers with regard to children’s play. Using perspectives from psychology, urban design, and cognitive science, we analyze the relationships between the design of built environments, parental attitudes and beliefs, and parental decisions on allowing children to play. For example, can a new implementation of children-centered urban design change parents’ skeptical attitude toward play? By drawing from global studies, we chart (1) the three key beliefs of parents regarding play and built environments: play should benefit learning, be safe, and match the child’s competence and (2) the design principles that can foster these beliefs: learning, social, and progressive challenge designs. By making the link between parents, urban design, and play explicit, this paper aims to inform parents, educators, policymakers, urban planners, and architects on the evidence-based measures for creating and increasing opportunities to play.

## 1. Introduction

Research on play offers clear evidence that play is critical for healthy growth, as play fosters children’s physical, social, and cognitive development [1,2,3,4,5]. For example, play fosters children’s theory of mind development—the ability to understand that others have minds and beliefs that may be different from one’s own, which is crucial in language and social interactions [6,7,8,9]. Preschoolers talk about math concepts while playing [10], developing both their verbal and numerical abilities. However, ask parents whether they think that play is a necessity—rather than simply “good to have sometimes”—and a different picture emerges. The idea that play can help children learn math, social skills, or language learning is probably not the typical notion that parents have in mind [11,12,13].

Why does this matter? Because parents’ ideas about play directly impact their children’s ability to play, either alone or with peers, in and outdoors. As decision-makers for their children, parents largely decide which activities their children can and cannot do, including play activities. If play is thought to be relatively useful or important, parents may create play opportunities for their children, such as making time to go to children’s museums [14,15,16]. On the other hand, if parents think that “it’s just child’s play”, then they may relegate play to times when nothing else of importance occupies children, or worse, simply forbid it. Indeed, researchers have raised great concerns regarding the lack of play in childhood (e.g., [17]), which has prompted wide-scale efforts to reverse the situation, from the reintroduction of recess times in school [18] all the way to redesigning cities to create play opportunities [19].

Here, we focus on urban design to foster children’s play because it is an ongoing mega-scale effort in large parts of the world. UNICEF launched the “Child Friendly Cities” (CFC) initiative in 1996, aiming to promote the creation of urban environments—such as building quality public playgrounds—where children can play, learn, and grow [20]. To date, the Child-Friendly Cities initiative has been launched in 38 countries and continues to grow. Similar initiatives have been launched by large nonprofit organizations, such as the Urban 95 initiative from the Bernard van Leer Foundation [21]—seeing cities from a height of 95 cm, which is the average height of a three-year-old. The Lego Foundation recently announced the “Build a World of Play” challenge [22], awarding DKK 900 million for proposals (and implementations) to create transformative environments of play. National and municipal governments have also focused on creating child-friendly urban environments [23]. For example, China put Child-Friendly Cities (an initiative similar to but independent from UNICEF) under their 14th Five-Year National Plan, mandating the building of 100 child-friendly cities. Given the enormity of resources poured into creating child-friendly urban spaces, current initiatives are in dire need of systematic input on how the intended users—children, parents, and educators—could truly benefit from these initiatives. Specifically, urban design that attempts to be child-friendly will not be effective in doing so unless parents perceive them to be. For example, no matter how sophisticated or lavish a playground design is, if parents think of the playground as unsafe, they will not allow their children to play in it. As such, urban design must be informed by evidence and an analysis of parents’ perceptions about urban environments and play.

This paper aims to provide such evidence and analyses by taking a novel perspective of focusing on parents rather than on children. This is because while children are the main beneficiaries of child-friendly urban design, as discussed above, it is mostly their parents who decide whether or not they get to play. In fact, a plethora of evidence from developmental science clearly shows that children are naturally curious and exploratory; as ‘little scientists’, they are keen on testing hypotheses about their environments [24,25]. Children do not need much encouragement to play; rather, it is their parents that need convincing. Thus, in order to reap the maximal benefits of child-friendly urban design, we need to understand how to satisfy parents’ needs as well as how to change parents’ perceptions of play for the better. In Section 2, we review the evidence showing the unique benefits of play—how play compares to other types of children’s activities, such as schooling—in the physical, social, and cognitive domains. Section 3 analyzes why, despite these benefits, parents tend to limit children’s play. This analysis allows us to chart the direct links between urban design, parents as decision-makers, and play opportunities—Section 4. Finally, in Section 5, we offer informed, evidence-based design principles for how to build child-specific urban environments.

## 2. Play Benefits Physical, Social, Emotional, and Cognitive Development

The first thing that comes to mind when thinking about the benefits of play is perhaps the physical domain; children get healthier from physical activities during play. Research backs up this notion; for example, children who spend more time outdoors playing have a lower body mass index (BMI) compared to those who spend less time playing outside [26]. Increased playtime is associated with a lower risk of being overweight [27]. Interestingly, children do higher levels of physical activity during games (children’s self-initiated play with rules, such as “hide and seek”) than they do during sports lessons with direct instruction [28].

But the benefits of play are not limited to the physical domain; instead, they extend to emotional, social, and cognitive development. Importantly, many of the benefits of play are unique: it is precisely the unstructured nature of play—as opposed to the structured nature of formal instructions and classrooms—that allows children to reap specific learning benefits. A prime example of this is executive function (EF), also commonly called cognitive control, which is the process of regulating thoughts and actions in support of goal-directed behaviors [29,30]. For example, when sitting in a classroom, a child may have to control his impulses to look outside the window so he can focus on absorbing what the teacher is saying. It is a common classroom woe that students get reprimanded for not being able to “concentrate”. Yet schools and classes do not teach EF skills per se. Studies have shown, however, that game activities can increase EF [31]. For example, preschoolers who play EF-like games during class time, such as “red light green light” (where they have to inhibit different reactions for each light color), increase their EF skills [32]. This is most likely because the unstructured nature of play creates opportunities for children to monitor their own thoughts and actions in order to achieve the goal of the game. For example, when playing hide and seek, one needs to control the impulse to laugh so as to not give away one’s location.

Play also confers social learning benefits by offering ample opportunities for children to learn how to negotiate and collaborate with others [2,33]. For example, it takes two to play see-saw, balancing and co-ordinating with each other. Role play—pretending to be a mom, a teacher, or a doctor—puts children in someone else’s shoes, facilitating their perspective-taking ability [34,35,36]. For example, when role-playing as a Mom, a child has to think about how to comfort her crying little doll, something that she ordinarily does not do in her actual role as a child. Indeed, perspective-taking ability relates to the ability to sympathize with others [37,38]. Just like EF, this crucially valuable skill is not taught in schools, yet children need to learn it somehow.

Not only does play foster these general “soft” skills, but it also supports verbal skills and academic learning. For example, children learn to utilize complicated language during play as they have to negotiate and co-ordinate with many different play partners, including new acquaintances [39,40]. Studies show that the more types and times children play, the higher their reading and writing abilities are [41,42]. In one study, two groups of four- and five-year-olds spent 30 min learning a list of new words, except that one group also spent a third of this time playing. While both groups made progress in their vocabulary understanding, only the children who played made progress in vocabulary expression—producing and using the new words, which shows better overall learning [43]. In yet another academic domain, play fosters mathematical learning because children naturally use math concepts during play [10]. One study on math and play found that a two-week intervention via playing number-related board games improved preschoolers’ counting and calculating abilities, particularly those from low-income families [44].

Because children have to act as their own agents during play—deciding what, how, where, and with whom to play—they become active learners [45,46]. In fact, at an exploration stage, where there are unknowns and uncertainties in the environment, the play does not differ from learning; one has to decide how to approach the problem at hand, whether it is about how to go up a seemingly difficult climbing wall or to solve a math problem. Exploration results in learning; one study showed that four- and five-year-old children learn geometric shapes better when they are given opportunities to explore, compared to those who are directly taught about the shapes [47]. Active learning can be especially helpful in a pedagogical context where teachers’ assumptions of students’ knowledge differ from students’ actual knowledge [48]. This mismatch can create situations where teachers select examples and materials that are hard for students to understand. But active learners can mitigate this situation by generating and selecting their own examples. Overall, the literature reviewed above shows that play not only directly results in cognitive, social, linguistic, and academic learning benefits, it also nurtures good learning habits—fostering one to actively search for knowledge and solve problems.

## 3. Why a Lack of Play?

Despite these enormous benefits of play, the current generation spends less time playing, especially outdoors [17]. This trend is observed globally, including in the US, Norway, England, and China [49,50,51,52,53,54]. One study found that in the US, outdoor play decreased by 25% between 1981 and 1997 among children aged 6 to 8 [55]. Surveys found that children played outdoors less than their mothers had in their childhood [56,57] and that mothers believe this lack of free play is detrimental to childhood development [58]. In kindergarten and elementary schools, free play has given way to more structured and educational activities [59,60].

The COVID-19 pandemic further diminished children’s playtime as digital games and social media took up the time that used to be spent on physical play [61]. Although parents spent more time at home during lockdowns, this time did not translate to more play time, mostly because parents have increased responsibilities such as working from home and homeschooling [62].

This decrease in play negatively impacts development. Even before the pandemic, researchers observed the trends of decreasing free play and rising obesity among children [63]. Recently, health concerns, together with the lack of outdoor playtime—including an increase in sedentary activities and sleep disruptions among children—were found worldwide in Italy [64], South Korea [65], China [66], and the United States [67].

### Why Parents Limit Play

What are the causes of reduced play? The popularity and ubiquity of digital media are often cited as the major culprits [51,56,68]. For example, the presence of TV, personal computers, or game consoles in children’s bedrooms is correlated with lower physical activities [69]. Another reason is the lack of availability, accessibility, and quality of outdoor play structures [70,71]. Indeed, parents around the world—including Greece [72], Wuhan, China [73], and Dhaka, Bangladesh [74]—think that their cities lack play infrastructure.

Clearly, one solution is simply to increase the quantity of play infrastructure. Indeed, UNICEF’s Child-Friendly City initiative places a significant emphasis on the provision of play spaces for children. For instance, cities such as Copenhagen [75] and Denver [76] have been recognized as CFC cities due to these cities’ provision of a high quantity of playgrounds that are high in quality. But while the provision of play infrastructure is a necessary step, it is not sufficient to increase play opportunities. For example, studies found that parents from different socioeconomic statuses (SESs)—determined by a combination of social and economic factors such as income and amount and kind of education [77]—display differentiated patterns regarding the utilization of urban environments. In particular, the same city facilities are utilized by low SES parents less than they are by higher SES parents, partly due to a lack of knowledge [78]. This lack of knowledge is probably associated with the primary factor that influences parental playground usage (allowing their children to play): beliefs about play.

Indeed, studies show that parents who believe that play is important for their child’s development play more frequently with their children compared to parents who do not hold this view [79,80,81,82,83]. This pattern is quite universal, found among Korean, Korean-American, Mexican, Indonesian, and Euro-American caregivers [84]. The belief can also be context-specific. For example, parents’ belief in the benefits of nature fosters children’s bond with the natural environment [85]. In contrast, when parents themselves think little about nature or are unfamiliar with it, they are less likely to expose their children to the natural environment [86].

The key to increasing opportunities to play, then, is to leverage the relationship between urban design and parental beliefs and perception. On the one hand, parental beliefs create the impetus for parents to utilize an urban environment. For example, belief in learning by exploration may lead parents to bring their children to museums [87]. On the other hand, urban design itself can change parents’ perceptions and beliefs about the importance of play. For example, the creation of pop-up playscapes in the city of Philadelphia [88] made parents reflect on their own childhood play times and on the importance of making time for their children’s play. Thus, urban design efforts, such as the Child-Friendly Cities initiative, must be planned and understood from the parent’s perspective. As such, what are the critical beliefs that underlie parents’ *positive* ideas about play? In the next section, we characterize these beliefs by analyzing situations where parents actually encourage their children to play.

## 4. Why Parents Allow Play: Three Key Beliefs

Examinations of play activities reveal that there are three key critical beliefs that induce parents to encourage rather than limit play. First is the belief that play offers learning opportunities—that play is not merely for fun. This is why, for example, parents are keen on taking their children to museums because they believe that children learn through play in the museum [89,90]. Second, parents need to believe that play is safe. Even if parents see learning benefits from outdoor play, they may still restrict their children from playing outside due to safety concerns [91]. In fact, parents, at times, allow digital play—which they view as offering little learning value—because they can monitor children’s location and safety [57,92]. Third, parents’ belief in their children’s competence and/or personality can influence parents’ decision to allow their children to play. For example, safety is relative to (parents’ perception of) children’s physical and cognitive competence: parents of older children may allow outdoor play because they are confident in their children’s ability to handle potential problems. Alternatively, parents who believe that their children are the “indoor” or “quiet” type might think that not much benefit is gained from outdoor play [93]. We discuss each of these three beliefs in greater detail.

### 4.1. Belief 1: Play Should Benefit Learning

When parents plan their children’s activities, they often consciously consider the learning benefits that children will derive from them [94]. Indeed, this belief in learning benefits is a primary reason for the rise in popularity of museum visits by families with young children [87,95]. Parents who visit museums with school-aged children emphasize the educational and learning functions of museums [96], particularly the importance of playful and hands-on encounters, multisensory explorations, and the fact that children interact with parents/guardians during museum visits [97]. Museum visits are also popular because parents believe that many of the resources offered by museums are not available at home or in other environments [98].

Another popular destination for parents to take their children is the zoo. Here, too, learning is most often cited as the benefit [99]. A survey of 1546 visitors to 13 zoos and aquariums found that, although visitors gave the highest priority to the entertainment aspects of the experience, they considered education about conservation to be an important aspect of a zoo or an aquarium visit [100]. Similarly, over 170 zoos across 48 countries report that the majority of their visitors come to learn [101]. Parents perceive going to the zoo as a great way to learn while having fun [102,103].

Parents also send their children to summer camps—another very popular activity—because they believe children will learn new skills and abilities at the camps. The skills range from specific things, such as music, drama, cooking, and knowledge about gardening, all the way to developing general social competence and selfconfidence by facing new adventures [104,105,106].

Notice that the learning benefits derived from museums, zoos, aquariums, and summer camps are the same learning benefits that children could get from daily outdoor play (reviewed in Section 2). So why do parents encourage museum visits but not play? The most likely reason is that museums, zoos, and camps are perceived to have clear learning content, while daily outdoor play does not. Indeed, even for museum visits, studies have found that some parents have difficulties articulating what their children learn specifically [95,107]. Making the learning benefits obvious, such as by providing written signage (of learning benefits), can change parents’ perception of the activity. For example, Song et al. [108] showed that, for the same exhibit, parents who saw the educational value signage (e.g., that the exhibit teaches math) rated the exhibit as having higher educational values in both academic and nonacademic areas when compared to parents who did not see the signage.

Parents’ belief in whether an activity carries learning benefits can also be easily influenced by advertisements. For example, in one study of parental toy choice [109], parents originally rated traditional toys as having higher educational values than electronic ones. However, after viewing advertisements about the (fake) learning benefits of electronic toys, parents changed their preference to favoring electronic toys. A similar pattern is found in parents’ behavior in downloading apps; the top downloads are those which clearly tout their learning benefits, regardless of whether clear evidence supports the claim [110,111]. This is in contrast to ordinary play, which—as we discussed in Section 2—carries clear benefits in physical, cognitive, social, and emotional development. What is lacking is parents’ *belief* that ordinary play confers these benefits.

### 4.2. Belief 2: Play Should Be Safe

Worries about safety during play are shared by parents all over the world and often become the decisive factor for whether or not children are allowed to play. Studies in the UK show that parents do not let children play outside because they believe it is not safe to do so [112,113], even if the playground is nearby their homes [114]. The same pattern is also found in case studies documenting parents’ usage of public spaces in Pune, India, Istanbul, and Turkey [115], as well as in Shanghai, China [116]. A study carried out in China showed that 89% of the children surveyed do not have the freedom to play outside due to caregivers’ concerns about safety, such as getting hurt during play [117]. Similarly, in a survey carried out in the US, nearly 19% of parents reported that they never allow their children to play outside. A majority of these parents cite safety concerns—traffic, violence, and drugs—as the reason for forbidding outdoor play [118].

Like beliefs about learning benefits, beliefs about safety are malleable. The physical environment, such as disorder, graffiti, and signs of vandalism, cue parents to believe that the area is not safe for their children to play [119,120]. On the other hand, the absence of traffic, such as in dead-end streets, makes parents perceive the environment as safe for play, even when there is no dedicated play infrastructure [121]. Sometimes, the physical cue that creates the perception of safety can be counter-intuitive: large amounts of data on human ratings of street-level imagery from the MIT Place Pulse dataset show that fences and walls are negatively correlated with perceptions of safety [122]. While fences and walls may be erected with safety in mind, in public spaces, humans often interpret them as markers of nonsafety: if the place is safe, then there is no need for a wall or fence in the first place. This counter-intuitive finding once again highlights the importance of considering human perception in design: sometimes, even when the design is intended to fulfill a function (e.g., security), it may bring out a different, even opposite, outcome in users’ perception.

Other than physical characteristics, the social variable (people) plays a significant role in parents’ safety perception. For example, collective efficacy—the degree to which neighbors trust and look out for one another—is positively associated with physical activity [123,124]. A higher maternal perception of a neighborhood’s collective efficacy is associated with more hours of outdoor play and fewer hours of television viewing [26]. At times, social cues can be more influential than physical ones: a comprehensive study of 10-year-old children’s outdoor activities in three US cities found that neighborhood social factors (e.g., collective efficiency and social contact) were the stronger predictors of physical activities compared to the neighborhood’s physical characteristics (e.g., traffic, physical disorder, etc.) [125].

### 4.3. Belief 3: Play Should Match a Child’s Competence

When making play-related decisions, parents consider not only the quality of the play (i.e., the safety and learning opportunities) but also the players, meaning their children’s competencies and personalities. The research found that when parents perceive children to be physically competent and sufficiently independent and when they have positive attitudes towards active free play, they arrange and support play activities accordingly [126,127]. As a result, children’s moderate-to-vigorous physical activities are correlated with parental perceptions of the children’s competence [128]. This leads to discrepancies in play opportunities. For example, parents allow boys to play outside at a younger age than girls [129].

Is parents’ perception of children’s competence and preferences accurate? As studies show, not necessarily so. For example, the self-reported social competence of children and adolescents differs from the report of their parents, teachers, or peers [130,131]. Parents also tend to overestimate physical risks and potential dangers. Interestingly, children themselves tend to be more accurate than their parents; interviews examining children’s understanding of play risks revealed that although four- and five-year-olds are not accurate in estimating the severity of injuries, their perception of risks and challenges tends to match their actual physical capacities [132].

What creates the discrepancy between parents’ perceptions and children’s actual competence is the fact that parents may not have many opportunities to observe their children play freely. Surveys found that whereas parents and children agree on how much time is spent on organized physical activities (e.g., sports clubs), children report more nonorganized physical activity than parents do [133]. Parents’ bias toward organized sports activities may influence their play decisions. For example, parents may decide whether or not to allow outdoor play based on their impression of how children like sports activities, even if the structure of organized sports could be different from that of free play. As yet another example of parent–child perception-competence discrepancy, a study found that the time parents spend helping with math homework is unrelated to their understanding of children’s math performance at school [134]. On the other hand, a study conducted in museums found that when parents watch their children play with peers and objects they do not have at home, parents gain a better understanding of children’s interests as well as their cognitive and social abilities [89]. In conclusion, in order to promote parents’ accurate understanding of children’s competence and personalities, we need to design outdoor play areas so that parents are able and willing to interact with and observe their children during play.

## 5. Design Principles

Our analyses of the key beliefs that can induce parents to allow and encourage play reveal two important things. First, the beliefs are interconnected: learning benefits alone are not sufficient if parents believe that play is unsafe, while safety is often relative to parents’ beliefs about their children’s competence. Second, these beliefs are changeable—some cues increase or create parents’ perceptions of learning benefits, safety, and their children’s competence, while some others may decrease these perceptions, thereby reducing children’s play opportunities. As such, in order to effectively design child-friendly urban spaces, the design must directly create or avoid these cues, responding directly to the parental needs and beliefs.

Connecting design to the users’ psychological needs is, of course, not a new concept; the literature on playground design is extensive, emphasizing how design must cater to the child playground users (e.g., see [135,136]). Broadly speaking, playground designers are encouraged to consider three things: (1) accessibility: ensuring easy access for all children, including those with disabilities, (2) high clarity: facilitating easy perception of the functionality of the design by users, and (3) safety management: minimizing the potential hazards and maximizing safety. For instance, designers may consider incorporating wheelchair-friendly rubber tiles to enhance accessibility, utilizing simple designs with familiar objects to promote high clarity, and incorporating age-appropriate challenges to support safety management. That is, the design is focused on optimizing positive experiences for children in playgrounds. 

But while these three design principles certainly have great merits, not all of them necessarily cater to the actual cognitive needs of children. For example, while high clarity seems to make sense as a playground design principle, the developmental science literature actually shows that an ambiguous, rather than an obvious, environment increases children’s curiosity and exploration [45,137,138]. Because the fields of developmental science and design/landscape architecture are separate, there tends to be a disconnect between what designers think children users need and the actual cognitive abilities of children (see [139] for a work connecting these two fields). More relevant to the scope of this paper, however, is that these playground design principles rarely consider the *parents* as playground users. As we have analyzed above, this consideration is even more critical as parents decide whether their children get to play or not.

To do so, in the final section of the paper, we consider concrete design principles that can effectively create or increase the three critical beliefs—learning benefits, safety, and the child’s competence—so parents become more likely to create play opportunities. We propose three design principles that can foster these beliefs: social design, learning design, and progressive challenge design. We select these design principles not to comprehensively cover the whole scope of child-friendly urban designs but rather to effectively and pointedly address the cognitive needs of parents as playground users and decision-makers.

### 5.1. Social Design

Social design refers to design that can promote a user’s social interactions, whether between the parents and children, among children, or even among the parents. The goal of social design is to evoke all three types of interactions, as each type contributes to creating parents’ beliefs around learning benefits, safety, and their child’s competence during play. First, the interaction between parents and children is cited by parents as one reason why they like taking children to museums, for its discovery and learning together aspect [107]. Likewise, the play between children also confers learning benefits, as children can learn from each other through imitation [140] or through social interactions and negotiations [33]. Importantly, when parents experience parent–child learning together or observe play among children, the parents are more likely to see the learning benefits. Second, the interaction between parents creates a sense of community and collective efficacy, which then leads to the perception of safety (Section 4.2). When parents know other adults, they feel safe to let their own children play [141].

**Figure 1:**
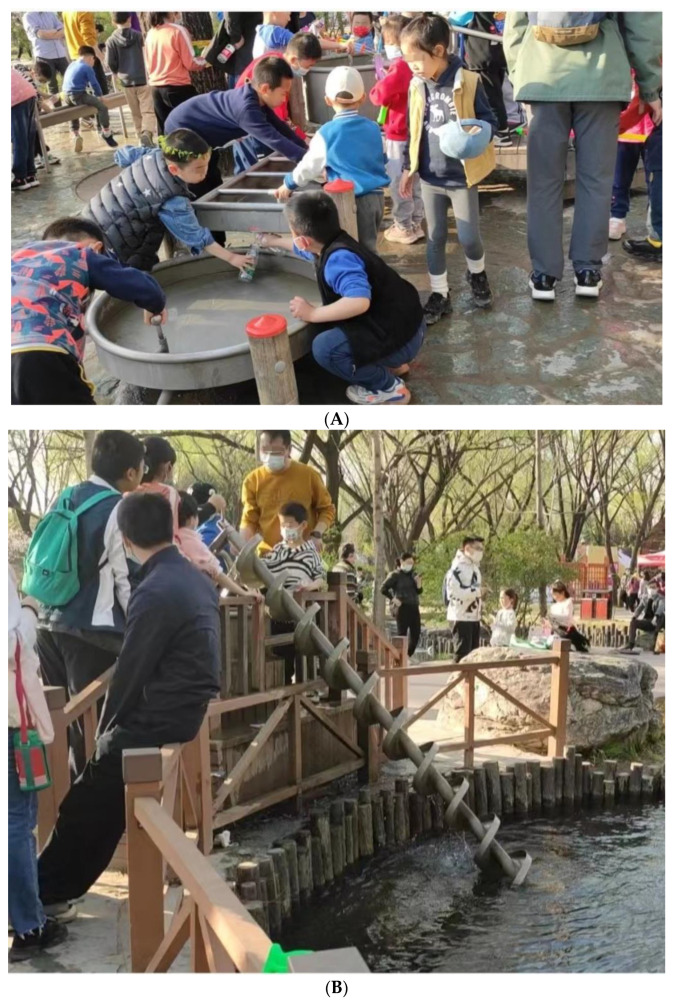
(**A**) An example of social design: a water device that requires cooperation from several people in order to play. Play installation in Haidian Park, Beijing, China. (**B**) An example of social design: a water intake shaft that cannot be used by a single user. Instead, a child must ask their parents or other children for help, creating parent-child and/or among children’s interactions. Play installation in Haidian Park, Beijing, China.

Social design, then, should directly consider not only children as the users but, importantly, the adults (the parents and guardians) as users. When design attracts parents as the users, the parents will be more likely to encourage play because they believe that such play confers learning benefits and safety and that their children are competent enough to play. This adds to and complements existing playground design principles that primarily focus on enhancing social interactions between children only rather than between children and parents [142]. In order to attract parents, we need designs that (1) afford physical togetherness and (2) afford challenges for users of different ages. An example of (1) is a swing with two face-to-face chairs that can be used by both parent and child or two children at the same time. An example of 2 is a water play installation that requires collaboration between several people in order to activate the device (see Figure 1A,B). This water device requires drawing water from the pool, which is labor-intensive and impossible for one child to complete alone. As a consequence, children ask parents or other children to help.

### 5.2. Learning Design

**Figure 2:**
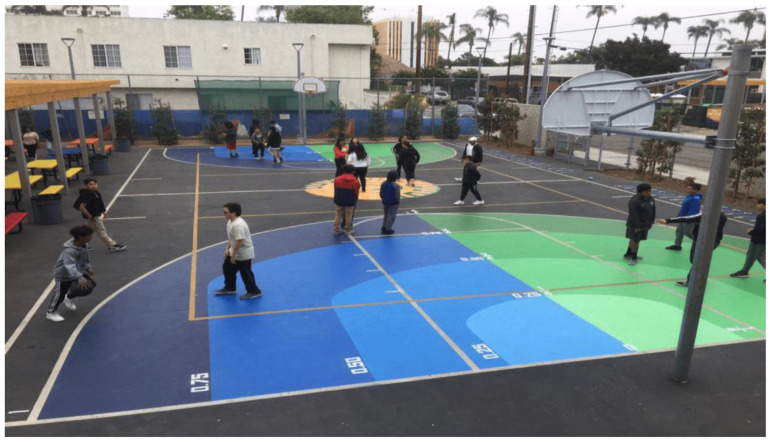
Example of learning design—a fraction ball playground in Santa Ana, CA. This is a basketball court that is painted with fraction lines, showing clear math learning while playing basketball. Created by researchers from UC Irvine and El Sol Sciences & Arts Academy [144].

As discussed in Section 4, parents care tremendously about learning benefits. They take children to museums or zoos because they believe that these activities offer clear learning benefits to their children, while they discourage children from playing outside because they think that mere play does not bring learning benefits, even interfering with learning. How can this belief be changed? By creating design that makes learning benefits salient for parents [108]. For example, drawing fraction lines on a basketball court (Figure 2) may make it salient to parents that children are learning mathematics while playing [143]. Excitingly, not only does such design make learning benefits visible for parents and teachers, a controlled study showed that it actually does increase children’s understanding of fractions [143].

**Figure 3:**
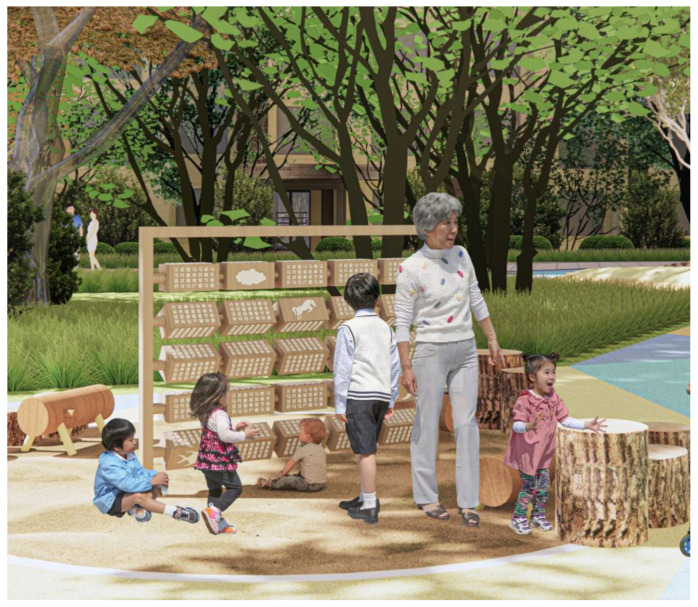
Example of learning design—Chinese Idiom Roller. In this game, the young and the old compete to say as many four-word-idioms (成语 cheng yu) as they can; the idiom must contain the pictured element (e.g., idioms containing the word horse). Original design by Stella Christie, drawn by Jianing Yin.

Another design concept that frontloads learning benefits is the creation of a teaching medium, enabling parents to teach something to their children by using the play installation. One example was created by our team, called the Chinese Idiom Roller (Figure 3). This play installation builds on four-word Chinese idioms (成语, cheng yu), which are explicitly taught at schools and used productively in both everyday and literary contexts. The play installation contains roller blocks with pictures of objects, for example, a horse. Players think up idioms that contain the said object, attempting to list as many as they can. The other sides of the roller contain many matching idioms so that players can compare their performance with a standard and perhaps learn new phrases. Grandparents can teach young ones about the idioms, or perhaps school children can teach/remind their parents of the idioms as they have just learned them at school. It is a social design game that confers clear learning benefits.

Design that externalizes learning benefits not only improves the perception that play results in learning but also reduces parents’ excessive attention to safety issues. Psychology studies show that people make decisions based on the most salient information rather than rationally considering all available information [145]. This applies to play decisions as well. For example, without salient learning benefits, parents may think that rock climbing is not safe. However, if the climbing wall contains salient math information—such as angles and distances of the rock climbing routes—parents may focus on the learning benefit, allowing their children to play.

### 5.3. Progressive Challenge Design

**Figure 4:**
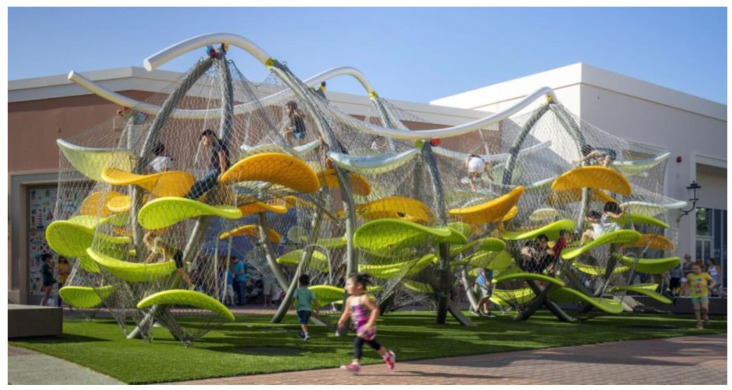
Example of progressive challenge design—Luckey Climber, at the Irvine Spectrum Center, Irvine, California, USA. The climber is afforded many levels of challenges such that the different physical abilities of the users can play together, and players can progress with experience [146].

A common child-friendly design principle is to design infrastructure based on age. For example, a short, easy-to-climb slide for toddlers and a higher, more challenging slide for older children. Although this design has some merits—children of different ages have different body sizes and physical abilities—an age-segregated design may actually reinforce parents’ belief that play is just mere fun. Instead, play installations that afford *progressive challenges*—from easy to difficult challenges within one installation—can foster parents’ perception of children’s competence. Take Luckey Climbers (Figure 4) as an example. This climbing maze challenges children to use their physical ability and spatial thinking to find a way to climb to the very top in a safe, enclosed space. Within one installation, novice climbers can initially set their target as the first or second level only and increase their target climb to as high as 15 feet from the ground as they gain more experience playing. This way, players can progress in their play, and, importantly, parents who observe such progression may gain confidence in their children’s competence to play. As we discussed in Section 4, parents’ perception of their children’s competence can greatly influence the likelihood of them allowing the children to play.

Another way to create progressive challenges is to design play infrastructure containing loose parts—stuff or objects—that children can move around themselves. Loose parts have been found to increase children’s engagement; for example, Barbour [147] reported that the inclusion of loose parts in play environments was associated with longer play duration. This is because loose parts—like sand in a sandbox or leaves and rocks in nature—allow children to design their own play experience [148]. Studies show that this freedom to create during play affords positive social behaviors—such as co-operating with others to build a sand tower—as well as creating new challenges for themselves [149].

**Figure 5:**
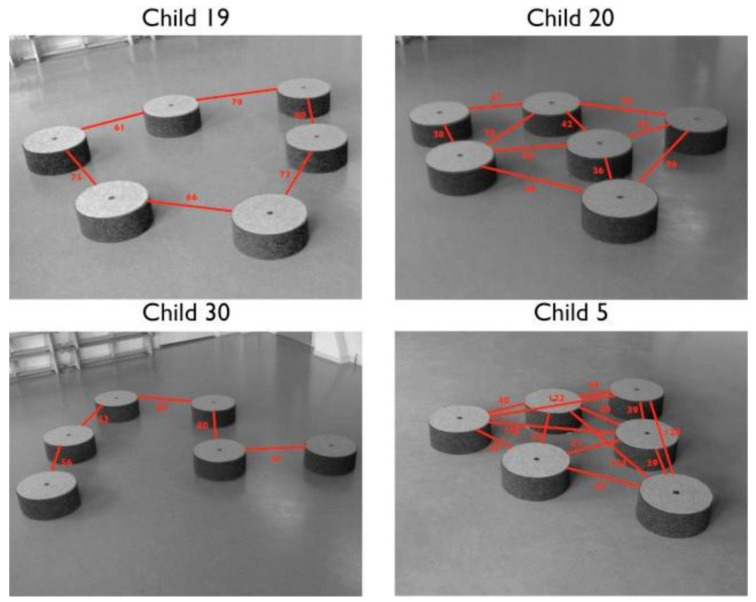
Example of progressive challenge design—playing with loose parts. In this game, children were free to arrange movable stone steps. The study showed that children arranged the stones based on their selfestimated jumping capabilities, creating their own challenges [150]. Figures reprinted with permission.

For example, one study showed that, in a jumping stone game (where the stones can be moved by the children themselves, Figure 5), children are capable of creating challenges that match their physical abilities [150]. In this study, children created various gaps between the stones according to their perceived maximum jumping distance. We suggest that such play not only fosters children’s cognitive development—a better estimation of selfability—but also creates opportunities for parents to observe their children’s competence.

## 6. Summary and Conclusions

How can play opportunities be increased? Our review focuses on a key yet under-analyzed factor in play: parents as the decision-makers. While there is abundant evidence that play benefits physical, cognitive, social, and emotional development, parents do not view play as important. Urban design can change this view, inducing parents to encourage rather than limit play. In order to do so, child-friendly urban design must consider parents’ beliefs rather than only focusing on the child users.

We identified three key parental beliefs that underlie parents’ positive ideas about play: the belief that play should carry learning benefits, that it should be safe, and that children must be competent enough to play. These three beliefs are interconnected in that learning benefits alone are not enough to make parents allow their children to play if they view the play as unsafe. In turn, parents’ belief about safety is relative to their perception of their children’s competence.

In order to aid policymakers, urban planners, and architects in creating environments that are conducive to play, we formulated three design principles that promote positive parental beliefs about play. Social design supports social interactions within play, which promotes the perception of safety and learning benefits. Learning design makes learning benefits explicit. Progressive challenge design allows children to set their own realistic and aspirational goals, which allows parents to gauge their children’s competence and observe their progress.

These three design principles not only address the previously ignored factor in play opportunities—that parents are the decision-makers—but also work in tandem with many existing principles and goals of the Child-Friendly Cities initiative. For example, CFC considers a clean environment—litter-free, reduced air and noise pollution, nontoxic surroundings, etc.—as a core requirement for creating child-friendly environments [151,152,153]. Clearly, a clean space is much easier to be perceived as safe by parents, which, in turn, is more likely to foster social interactions between the parents and children. Other CFC design themes, such as Social Connection and Learning [154], are also aligned with the social design and learning design that we propose here. However, different from prior works, we believe that it is critical to highlight and specifically target parents’ perceptions.

Last but not least, policymakers and taxpayers alike may wonder whether we need to purposefully build child- and parent-friendly infrastructure rather than having free, open spaces. While some free, open spaces are clearly needed, our evidence-based analysis shows that mere open spaces will not sufficiently assuage parental negative bias about free play. That is, if the goal is to increase play opportunities, we need to build purposeful design infrastructures. To reiterate our point with yet another example, despite the demonstrated benefits of natural green spaces to children’s health and well-being [155] and its ubiquitous mention in child-friendly environment literature (e.g., [154], parents still harbor reservations about allowing their children to play in such settings due to perceived safety concerns [91].

Urban environments are vital to shaping children’s physical, cognitive, emotional, and social development. Among other factors, urban environments provide quality play spaces, and play is one critical medium through which children grow and develop. In this paper, we conclude that it is not adequate for urban design to merely consider children as the only space users because play opportunities are usually directly determined by parents. Urban design, then, must seriously consider parental needs, especially since the parents of the current era tend to be cautious about their children’s daily activity arrangements and whereabouts and are less likely to allow unsupervised free play [91]. We believe that adopting the design principles proposed here will assuage parental concerns and, as a result, increase children’s opportunities to play.
